# The Selective Inhibitory Effect of Hyperthermia on the Metabolism and Growth of Malignant Cells

**DOI:** 10.1038/bjc.1971.91

**Published:** 1971-12

**Authors:** D. S. Muckle, J. A. Dickson

## Abstract

**Images:**


### THE SELECTIVE INHIBITORY EFFECT OF HYPERTHERMIA ON THE METABOLISM AND GROWTH OF MALIGNANT CELLS

D. S. MUCKLE AND J. A. DICKSON

*From the Department of Surgery, University of Newcastle upon Tyne, and Cancer Research Unit, Department of Clinical Biochemistry, Royal Victoria Infirmary, Newcastle upon Tyne*

Received for publication August 10, 1971

**SUMMARY.—Hyperthermia (42° C.) exerted an inhibitory effect on the $O_2$ uptake of rabbit VX2 carcinoma cells *in vitro*, and led to a decrease in viability and growth potential of the cells, as measured by their ability to produce tumours in rabbits. Anaerobic glycolysis of the tumour cells was unaltered by hyperthermia. Respiration and anaerobic glycolysis of normal rabbit liver, kidney and red blood cells were unaffected by the elevated temperature. Local heat was applied to established VX2 tumours *in vivo*, with a subsequent reduction in tumour size in all cases, the most effective therapy regime being 3 one-hour applications of heat within the mean cell generation time of the tumour. Following heating there was rapid and widespread tumour cell necrosis and lysis, with subsequent replacement of the tumour architecture by connective tissue. There was a prolongation of survival time in 50% of the treated animals, which are still alive 18 months after therapy; all the control animals died within 10 weeks. The selective inhibitory effects of hyperthermia on cancer cells, and its application to human neoplasms, are discussed.**

RECENT interest in the use of hyperthermia (body temperatures of 40° C. and above) as a clinical adjuvant to cancer therapy, (for example, the *B. coli* " auto-vaccine " included in the basic treatment regime at the Ringberg Klinik—see Issels (1970)), revives the original observations of Busch (1866) and other workers (Bruns, 1888; Gerster, 1892), who noted that patients suffering from neoplastic disease often had a regression of both primary and secondary tumours following an attack of erysipelas or other severe pyrexial infection. Remissions were described in patients with a variety of tumours including neoplasms of the breast, face, cervical region, malignant melanoma, penile tumour, periosteal sarcoma, lymphosarcoma, ovarian and uterine carcinoma. Coley in 1893 treated cancer patients with a " toxin " (consisting of a crude filtrate from a culture of haemolytic streptococcus and *B. prodigiosus*) and observed that sarcoma responded more readily to the induced hyperthermia than carcinoma. A critical and detailed case analysis of 30 inoperable and histologically confirmed tumours, which regressed completely following toxin therapy by Coley, has been published (Nauts *et al.*, 1953); there was recurrence of the cancer in only one of the patients, some of whom were examined at follow-up clinic for almost 50 years. Although Coley continued to use commercial preparations of his pyrogenic fluid for the treatment of cancer until his death in 1936, the toxin was rarely, if ever, standardized biologically and consequently its effects were inconsistent and capricious.

Successful treatment with the bacterial products led to tumour haemorrhage and necrosis, and in the early 1940s Shear and his group attempted to isolate the pyrogenic and tumour-necrotizing substance and devise a method of bioassay to standardize its potency (Shear and Perrault, 1944). The toxin was characterized as a high molecular weight polysaccharide, but its effect on transplantable sarcoma 37 in mice was too unpredictable for this animal system to be used for standardization. Although greatly purified preparations of bacterial polysaccharides capable of inducing pyrexia and tumour necrosis have been obtained (Creech *et al.*, 1948), the high toxicity of such extracts has discouraged extensive clinical studies (Reimann and Nishimura, 1949).

In 1968 a project was instituted to investigate the effects of hyperthermia on the metabolism and growth of the VX2 rabbit carcinoma. Respiration and glycolysis were studied as indices of cell metabolism, in association with a dye uptake test for cell viability, and tumour growth rate was followed by *in vivo* volumetric measurements. Externally applied heat has been employed to elevate tumour temperature, and the experimental animal system is being used to investigate the potential and possible application of this method in the treatment of primary and metastatic malignant disease in man.

### MATERIALS AND METHODS

The VX2 tumour is an anaplastic, squamous cell carcinoma that developed as a result of malignant change in the cells of a Shope virus-induced skin papilloma of a domestic rabbit (Kidd and Rous, 1940). The tumour is highly malignant, metastasizing to lymph nodes and lung but rarely to other sites. For 5 years after induction of the original VX2 carcinoma the blood of all rabbits bearing the transplantable tumour contained specific antibody to the papilloma virus; the antibody can no longer be detected in the blood of tumour-bearing rabbits, however, and it is not known whether the virus has disappeared entirely from the VX2 carcinoma cells or still persists in some non-antigenic form (Rous *et al.*, 1952).

The VX2 carcinoma was maintained by serial transfer of one million cells injected 1 cm. deep into the thigh muscles of male New Zealand white rabbits weighing 2·5 kg. and fed an *ad libitum* diet. Tumour volume was calculated from caliper measurements made in the antero-posterior, lateral and vertical planes of the leg, allowance being made for the animal's normal tissues on the basis of measurements made on the limb of each animal before inoculation of tumour cells. The tumour measurements were taken at weekly intervals and the results plotted as volume (c.c.) against time. This method compared favourably with log, semilog and cube root methods of assessing tumour growth (see Mendelsohn, 1963).

The tumour was excised from the rabbit's hind limb under nembutal anaesthesia (0·6 c.c./kg. body wt., IV) and placed in $Ca^{++}$- and $Mg^{++}$-free Rinaldini solution (Rinaldini, 1959) at 4° C. The tumour was finely minced, washed with cold Rinaldini solution to remove debris and blood cells, and placed in a 50 ml. flask with a magnetic stirrer. The tumour was then disaggregated into a unicellular suspension using 30 ml. Rinaldini solution containing 1% trypsin (Difco 1 : 250), 0·01% deoxyribonuclease (DNase grade II, Seravac) with its activator $Mg^{++}$ ($Mg\ SO_4$, 0·01%) and penicillin (100 units/ml.). After stirring at 37° C.

for 20 minutes in this solution, the suspension was filtered through an 80 mesh stainless steel sieve followed by a 200 mesh sieve and centrifuged at $200 \times g$ for 5 minutes. The cell pellet was washed in Waymouth culture medium containing 10% pooled human AB serum. The cells were resuspended in fresh medium, counted on a haemocytometer and assessed for viability using trypan blue dye (Hoskins *et al.*, 1956). By this method over 80% of the cells were viable at the commencement of each experiment. Rabbit kidney cells were obtained by the same technique, using 0·5% trypsin without DNase, and liver cells were obtained by a modified Jacob and Bhargava method for liver dissociation (Suzangar and Dickson, 1970).

For respiration studies, an optimal number of cells, $5–10 \times 10^6$, was suspended in 3 ml. of 0·02M Tris-HCl buffer, pH 7·4 containing 0·1M sucrose in each Warburg flask, with 0·2 ml. 10% KOH in the centre well. The $O_2$ uptake was expressed as $\mu$l. per mg. dry weight of tissue per hour ($QO_2$). Experiments were carried out simultaneously at 37·5° C. and at 42° C. on cell populations from the same tumour using independent water baths. Cells were removed at hourly intervals from the flasks and stained with trypan blue.

For measurements of anaerobic glycolysis, $5–10 \times 10^6$ cells were placed in each Warburg flask and the $CO_2$ production recorded. The suspending medium consisted of 3·2 ml. Tris-$HCO_3$ buffer, pH 7·4 (0·05M Tris plus 0·154M $NaHCO_3$), or 3·2 ml. Tris/$HCO_3$/glucose (2 g./l.) in alternate flasks. 95% $N_2$ and 5% $CO_2$ ($O_2$ content of mixture less than 20 p.p.m., Air Products Ltd.) was used as the gas phase. Experiments on cells from the same tumour were carried out at 42° C. with a control series of flasks at 37·5° C., as for respiration studies. Results were expressed as $\mu$l. $CO_2$ produced per mg. dry weight of tissue per hour ($QCO_2$). All manometric observations were carried out in duplicate (or more) flasks.

The *in vivo* studies were performed on anaesthetized animals, and heat was applied to an established tumour by immersion of the hind limb in a water-bath at 46° C. for 1 hour on 3 consecutive days, 5 weeks after inoculation. At this time the tumours were 3–4 cm. in diameter and relatively non-necrotic. The three consecutive heat applications fell within the estimated tumour cell generation time of 87 hours (Bullough and Laurence, 1968). Tumour, flank and thoracic muscle temperatures were measured throughout the experimental period using a Cambridge potentiometer (type 44228) with copper–constantan thermocouple needles, which are sensitive to temperature change only at the electrode (needle) tip.

### RESULTS

The $O_2$ uptake *in vitro* of VX2 cell populations from 8 different tumours was significantly depressed ($P < 0·001$) after 1 hour at 42° C. compared to the $O_2$ uptake in equivalent control populations from the same tumour maintained at 37·5° C. (Fig. 1). Normal rabbit liver, kidney and red blood cells showed no depression of $O_2$ uptake at 42° C. (all 3 tissues from 6 different animals were examined). Anaerobic glycolysis of the tumour cells (6 tumours studied) was unaffected by the increased temperature. At 42° C., there was a marked and progressive decrease in viability of the tumour cells with time, as assessed by dye uptake, and this decrease was significant after the first hour (Fig. 2). Cells heated *in vitro* at 42° C. produced no tumours when injected into the host if the cells had been maintained at this temperature for more than 2 hours, even though 50% of the VX2 cell population inoculated was still viable at this time. For this experiment $1 \times 10^6$ viable (unstained) cells were inoculated into 2 or more rabbits; heated cells from 6 different tumours were tested. Cell populations removed from the flasks after 1 hour were 70% viable and produced tumours that appeared later, and grew more slowly, than tumours induced in control animals by inoculation of cells which had been maintained in flasks at 37·5° C. for the same time. There was no morphological difference between tumours produced by heated or unheated cells.

FIG. 1.

FIG. 2.

FIG. 1 and 2.—The values plotted in Fig. 1 and 2 represent the mean ± standard deviation from experiments with tumours each from a different rabbit. The figures in brackets indicate the number of tumours studied at each point on the appropriate curve. Cell populations were heated at 42° C. and compared with equivalent control cell populations from the same tumour simultaneously maintained at 37·5°, using independent water baths. All procedures on cell populations from each tumour were carried out in duplicate.

Eight rabbits with established tumours (5 weeks after inoculation) were treated with local hyperthermia (tumour temperature 42–43° C., flank temperature 40–41° C.). There was a significant reduction in tumour volume ($P < 0{\cdot}001$) compared to 10 unheated tumours 2 weeks after heating (Fig. 3), and although 4 of the heated rabbits died after 10 weeks from lung metastases, the primary tumours were reduced in size at time of death by a mean of 60% compared with tumour size at 5 weeks.

There were rapid and striking changes in the histology of the tumours following heat treatment. In the untreated tumour the cells form sheets and irregular masses, separated by a fine connective tissue stroma (Fig. 4). The tumour cells are polygonal with deeply staining cytoplasm, and mitotic figures are numerous (Fig. 5). Twenty-four hours after the third heat application the tumour showed widespread necrosis, with pyknosis, karyorrhexis and cell lysis (Fig. 6). Intense fibroblast and macrophage activity then occurred (Fig. 7) with subsequent replacement of the tumour architecture by connective tissue, in which no cancer cells could be identified (Fig. 8).

FIG. 3.—Each point is the mean ± standard deviation for tumour volume in 8 heated tumours and 10 unheated control neoplasms.

### DISCUSSION

Hyperthermia produced selective and irreversible damage to the VX2 tumour cells both *in vitro* and *in vivo*. Cell respiration was inhibited after heating for 1 hour *in vitro*, and the cells failed to produce tumours after 2 hours applied heat. It was possible to produce a more rapid inhibition of respiration in the VX2 cells by temperatures above 42° C.

That 50% of the tumour cells remained unstained by trypan blue after 2 hours heating may indicate that the biochemical processes concerned with maintenance of membrane integrity in the VX2 cells are less sensitive to damage than is respiration under these conditions. A similar discrepancy between the results of bioassay and those obtained by dye exclusion has been reported recently for L1210 leukaemia cells subjected to 43° C. (Giovanella *et al.*, 1970). After 2 hours hyperthemia 15% of the cells were unstained, but injection of $1 \times 10^4$ heated cells into mice caused no deaths; in the L1210 leukaemia mouse bioassay system a single viable cell can cause death (see Giovanella *et al.*, 1970). It is of note that although the exclusion of vital dyes can be regarded as a reliable method for assessing viability in cell suspensions under normal conditions (see Dickson, 1970), the technique has been found to be of doubtful value for measuring growth potential of cells stored at low temperatures (Schrek, 1965). Interpretation of the results of dye uptake tests on cells subjected to extremes of temperature requires caution therefore, and exclusion of dye is not necessarily equated with cell viability.

Despite the oft-cited importance of both aerobic and anaerobic glycolysis in cancer cell metabolism (Busch 1962; Pitot 1966), and the specific positive correlation between anaerobic glycolysis and growth rate in hepatomas (Burk *et al.*, 1967), anaerobic glycolysis was apparently not related to the ability of the VX2 cells to produce tumours, since heated and control cell populations had similar $QCO_2$ values when inoculated into hosts.

The initial lesion and precise mode of action of hyperthermia on cancer cells has not been identified as yet, although several aspects of cancer cell biochemistry seem susceptible to damage by hyperthermia. High temperature inhibits the incorporation of $^3$H-thymidine into the DNA, and $^{14}$C-amino acids into the protein of tumour cells (Mondovi *et al.*, 1969). Polyribosome disaggregation with disruption of the translation process occurs in HeLa cells incubated at 42° C. (McCormick and Penman, 1969). Heat prolongs the lag phase and inhibits logarithmic multiplication in rat mammary gland cancer cells in monolayer culture, an early effect being depression of RNA synthesis (Dickson and Shah, unpublished work).

Three applications of local heat within the reported tumour cell generation time resulted in significant reduction in tumour size, with apparent cure of half the treated rabbits. That 50% of the treated animals still died from metastases may indicate that in these cases the tumour became disseminated early, before local heat was applied. It may also be that in the animals which had a favourable response to hyperthermia, destruction of the primary tumour enabled, or stimulated, the rabbit's immune system to cope with the metastases, while in the rabbits which died there was an inadequate (or no) immune response. The effect of general body hyperthermia on secondary, as well as primary, cancers is currently being investigated.

---

EXPLANATION OF PLATES

FIG. 4.—Untreated rabbit VX2 carcinoma. The tumour consists of sheets and irregular masses of cells in a fine connective tissue stroma. × 140.

FIG. 5.—VX2 carcinoma cells showing hyperchromatic nuclei with fine chromatin and one to several nucleoli. Three cells in mitosis are present, two in metaphase (centre field) and one in early prophase (top centre). × 800.

FIG. 6.—VX2 carcinoma 24 hours after third application of hyperthermia. The tumour shows widespread necrosis, with pyknosis, karyorrhexis and cell lysis. × 800.

FIG. 7.—VX2 carcinoma 4 weeks after heat treatment, showing fibroblasts and a group of macrophages (bottom right of field). × 800.

FIG. 8.—VX2 carcinoma 12 weeks after hyperthermia. The tumour architecture has been replaced by fibrous tissue and no cancer cells can be seen. × 140.

4

Muckle and Dickson.

5

6

Muckle and Dickson.

7

8

Muckle and Dickson.

Recent work in man has shown that hyperthermia, achieved by regional perfusion (Cavaliere *et al.*, 1967; Stehlin, 1970) or by total body immersion (von Ardenne and Rieger, 1967) can have a dramatic effect on tumour growth, producing such massive necrosis with bone fracture in limb tumours that amputation of the non-functional limb was required. Other severe complications have occurred including vascular collapse, myocardial infarction, second degree burns, and kidney failure due to necrotic tumour material entering the blood stream (Cavaliere *et al.*, 1967). The use of hyperthermia for the treatment of cancer, although of undoubted effectiveness in the cases reported to date, has involved considerable hazard to the patient. The VX2 carcinoma is a rapidly growing and metastasizing tumour which is a convenient model in which to study the effects of high temperature therapy for cancer, with a view to evolving a satisfactory method of inducing hyperthermia for application to human neoplasms. The value of combined hyperthermia, radiotherapy and chemotherapy is also under study.

The authors express their appreciation to Professor I. D. A. Johnston, Department of Surgery, for his enthusiastic interest in this project and for his criticism of the present manuscript; and to Mr. R. McCoy, Cancer Research Unit, for technical assistance. The work was supported by the North of England Council of the Cancer Research Campaign.

### REFERENCES

BRUNS, P.—(1888) *Beitr. klin. Chir.*, **3**, 443.

BULLOUGH, W. S. AND LAURENCE, E. B.—(1968) *Eur. J. Cancer*, **4**, 587.

BURK, D., WOODS, M. AND HUNTER, J.—(1967) *J. natn. Cancer Inst.*, **38**, 839.

BUSCH, H.—(1962) ‘ Introduction to the Biochemistry of the Cancer Cell ’. London (Academic Press), p. 313.

BUSCH, W.—(1866) *Verh. naturh. Ver. preuss. Rheinl.*, **23**, 28.

CAVALIERE, R., CIOCATTO, E. C., GIOVANELLA, B. C., HEIDELBERGER, C., JOHNSON, R. O., MARGOTTINI, M., MONDOVI, B., MORICCA, G. AND ROSSI-FANELLI, A.—(1967) *Cancer, N.Y.*, **20**, 1351.

COLEY, W. B.—(1893) *Am. J. med. Sci.*, **105**, 487.

CREECH, H. J., HAMILTON, M. A., NISHIMURA, E. T. AND HANKWITZ, R. F.—(1948) *Cancer Res.*, **8**, 330.

DICKSON, J. A.—(1970) *Expl Cell Res.*, **61**, 235.

GERSTER, A. G.—(1892) *N.Y. med. J.*, **1**, 641.

GIOVANELLA, B. C., LOHMAN, W. A. AND HEIDELBERGER, C.—(1970) *Cancer Res.*, **30**, 1623.

HOSKINS, J. M., MEYNELL, G. G. AND SANDERS, F. K.—(1956) *Expl Cell Res.*, **11**, 297.

ISSELS, J.—(1970) *Clin. Trials J.*, **3**, 357.

KIDD, J. G. AND ROUS, P.—(1940) *J. exp. Med.*, **71**, 813.

MCCORMICK, W. AND PENMAN, S.—(1969) *J. molec. Biol.*, **39**, 315.

MENDELSOHN, M. L.—(1963) ‘ Cell Proliferation ’, edited by L. F. Lamerton and R. J. M. Fry. Oxford (Blackwell), p. 190.

MONDOVI, B., FINAZZI, A., ROTILIO, G., STROM, R., MORICCA, G. AND ROSSI-FANELLI, A.—(1969) *Eur. J. Cancer*, **5**, 137.

NAUTS, H. C., FOWLER, G. A. AND BOGATKO, F. H.—(1953) *Acta med. scand.* Suppl. **276**, 1.

PITOT, H. C.—(1966) *A. Rev. Biochem.*, **35**, 338.

REIMANN, S. P. AND NISHIMURA, E. T.—(1949) *J. Mich. St. med. Soc.*, **48**, 453.

RINALDINI, L. M.—(1959) *Expl Cell Res.*, **16**, 477.
ROUS, P., KIDD, J. G. AND SMITH, W. E.—(1952) *J. exp. Med.*, **96**, 159.
SCHREK, R.—(1965) *Cryobiology*, **2**, 122.
SHEAR, M. J. AND PERRAULT, A.—(1944) *J. natn. Cancer Inst.*, **4**, 461.
STEHLIN, J. S.—(1970) 'Surgical Oncology', edited by F. Saegesser and J. Pettavel. Vienna (Hans Huber Publishers), p. 859.
SUZANGAR, M. AND DICKSON, J. A.—(1970) *Expl Cell Res.*, **63**, 363.
VON ARDENNE, M. AND RIEGER, F.—(1967) *Z. Krebsforsch.*, **69**, 341.


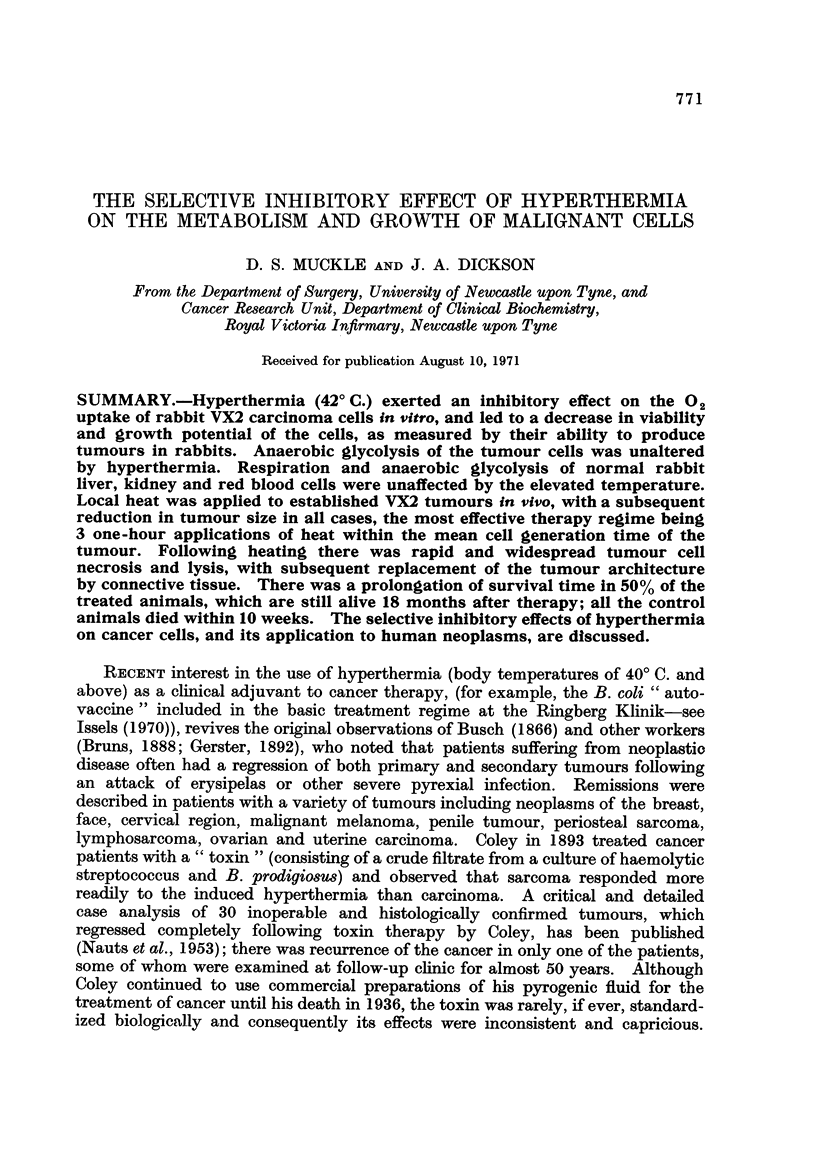

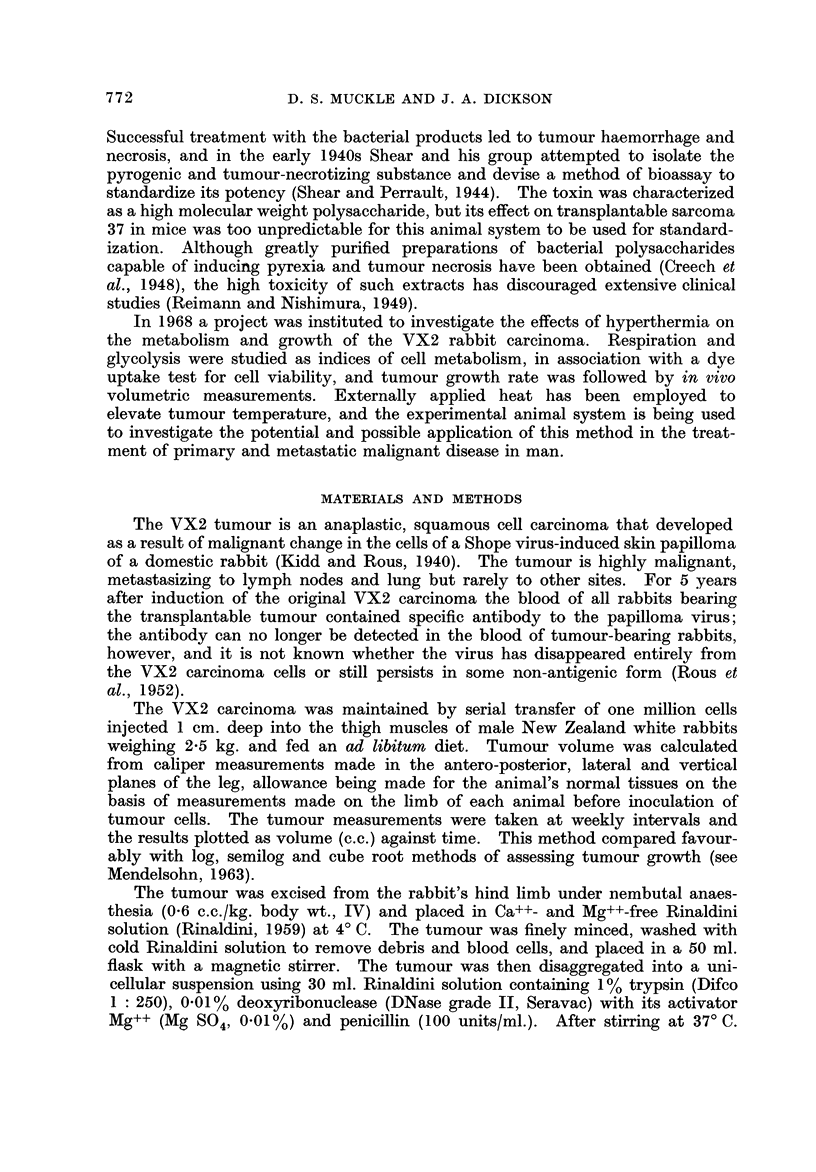

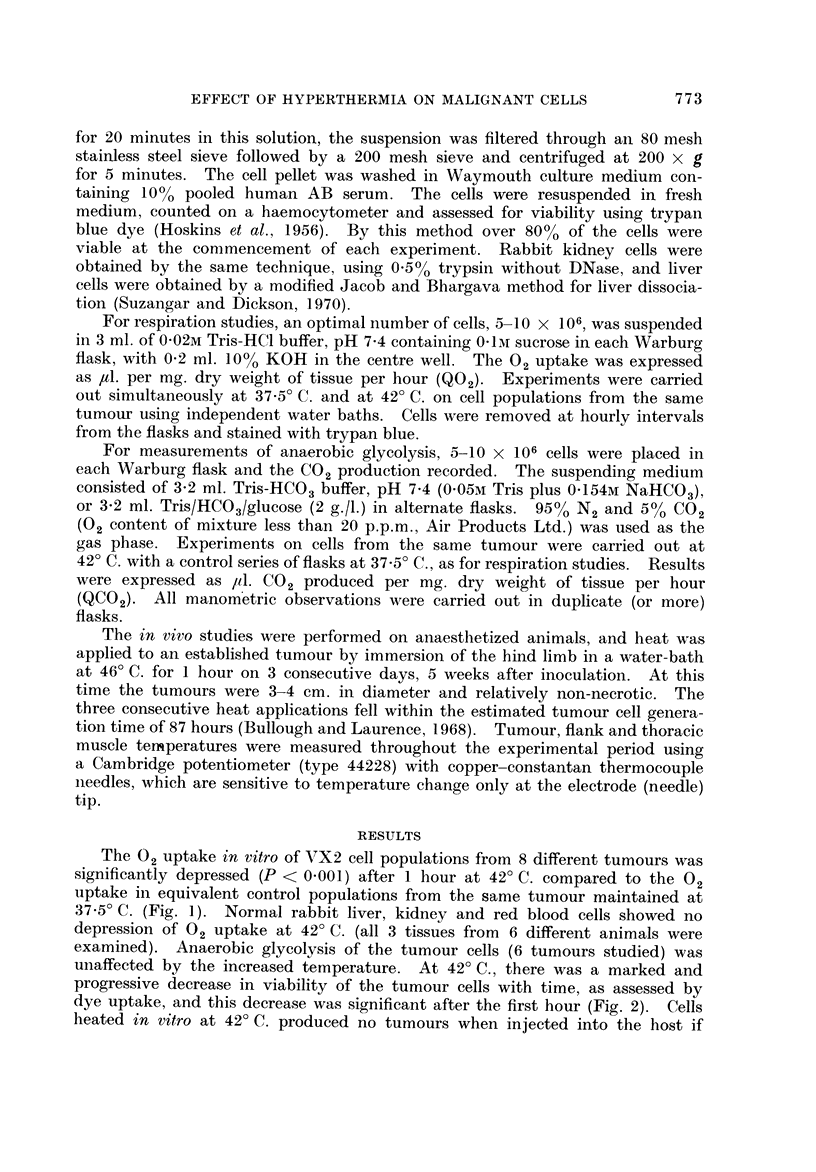

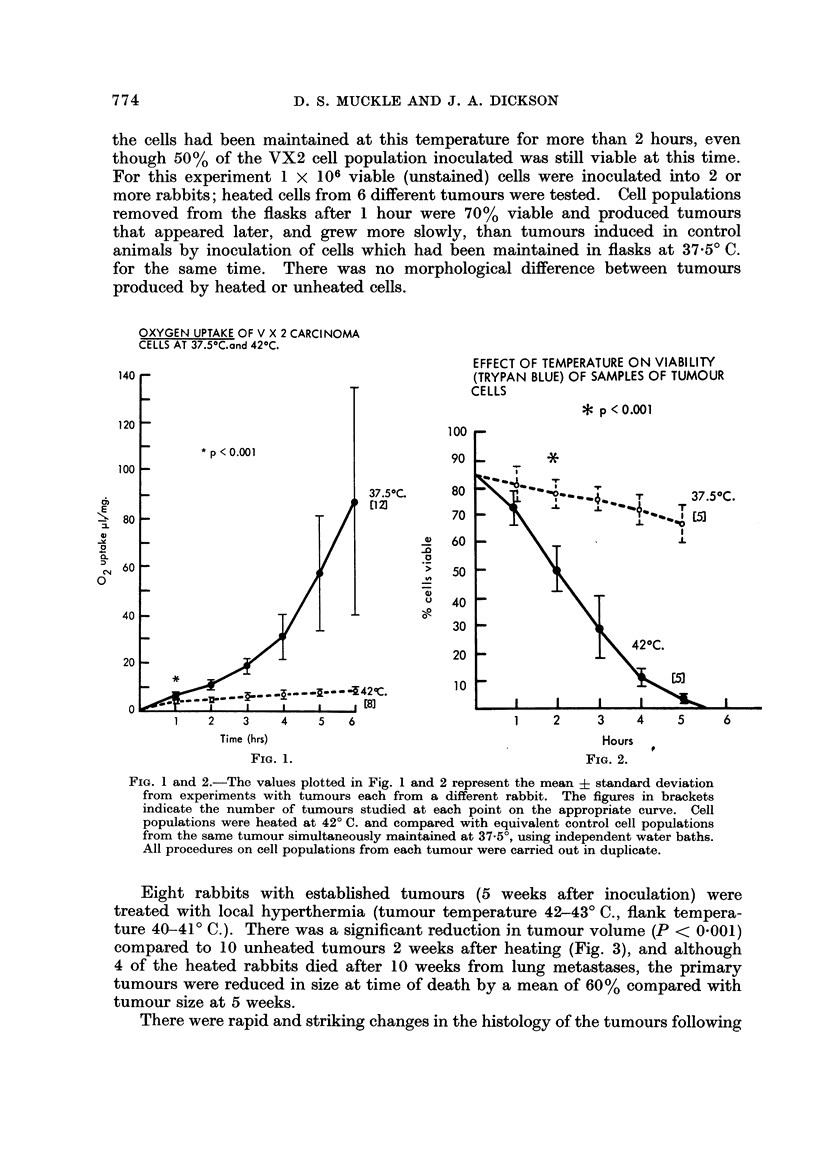

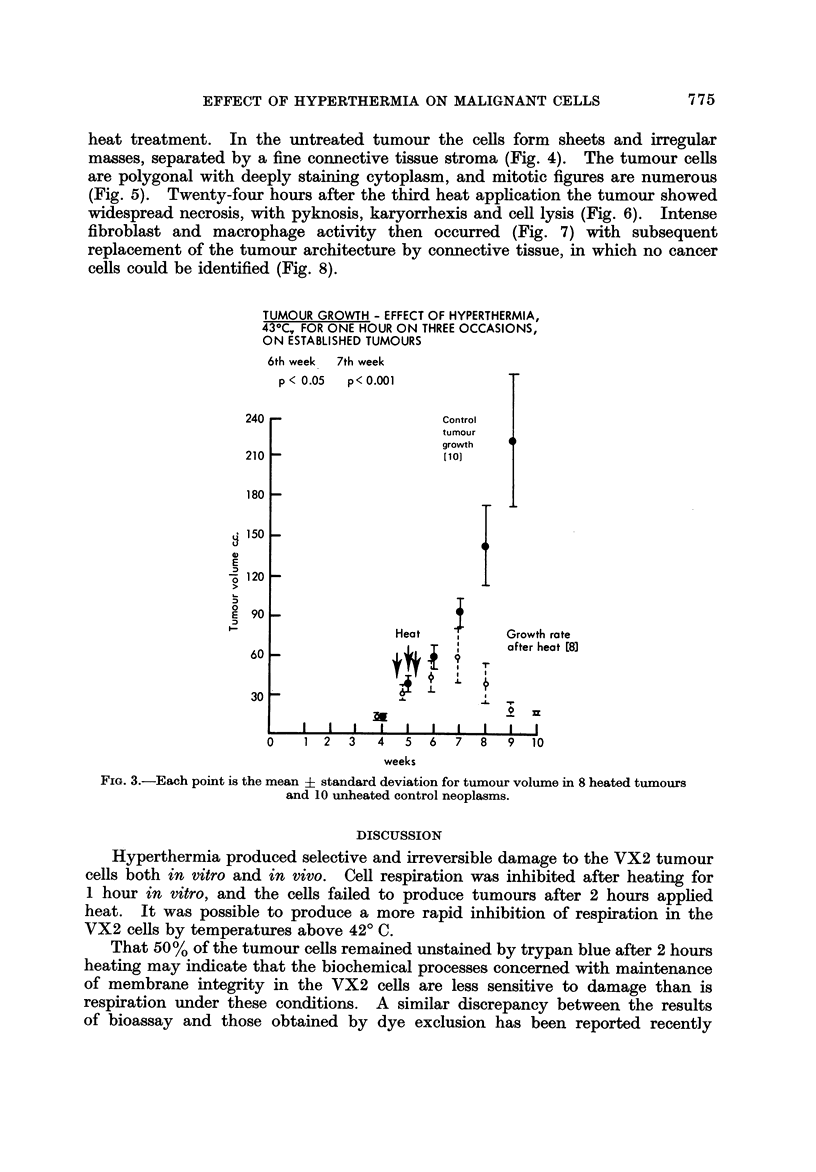

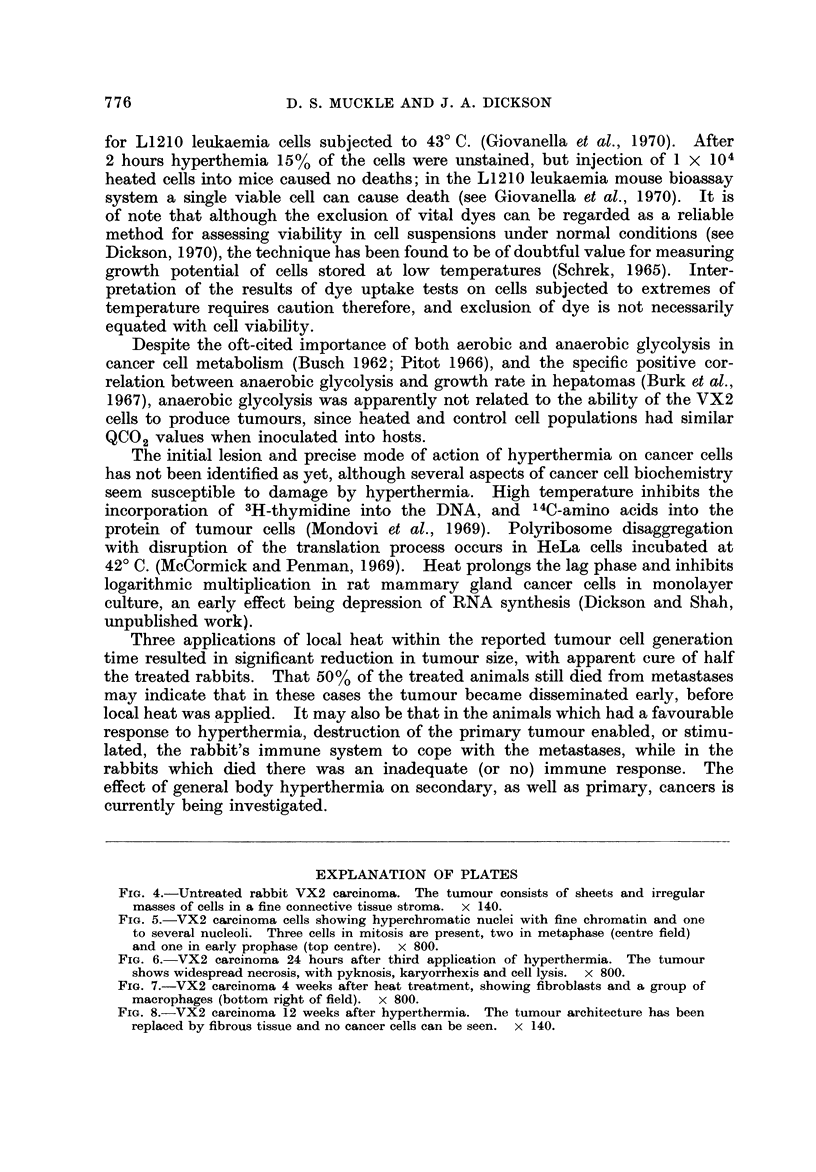

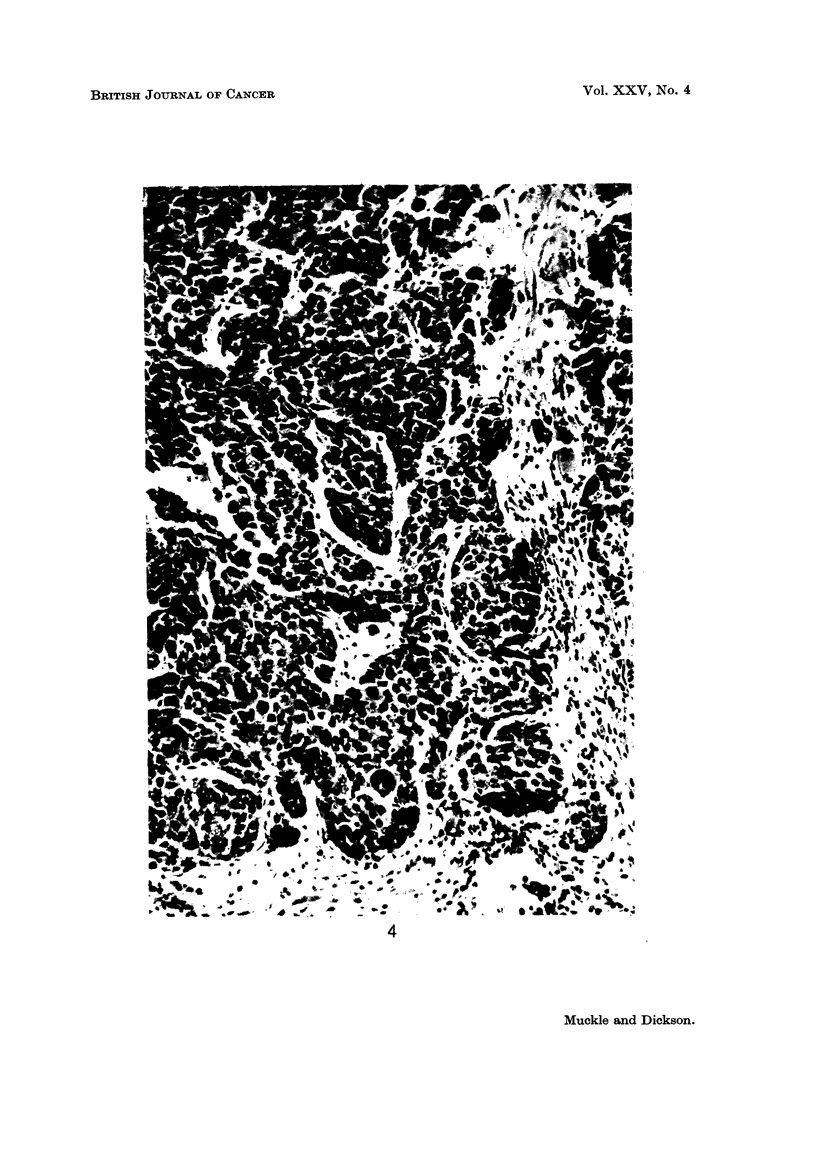

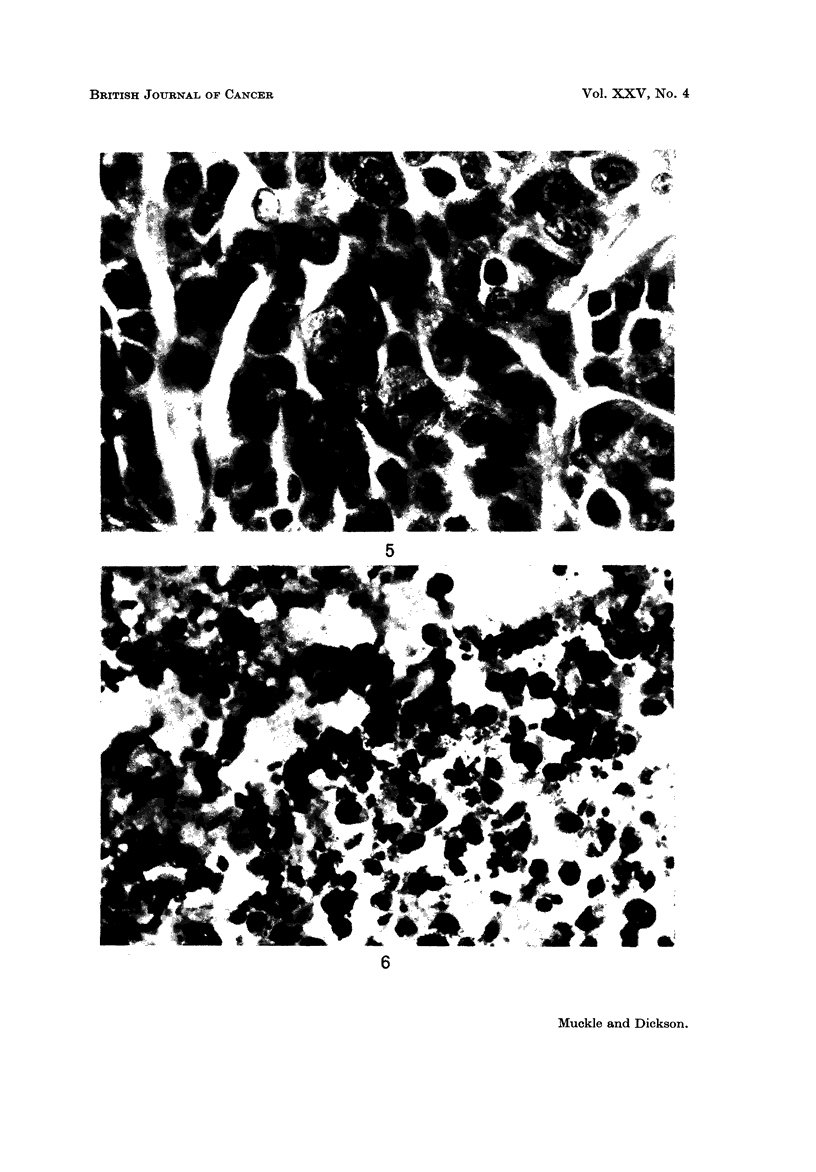

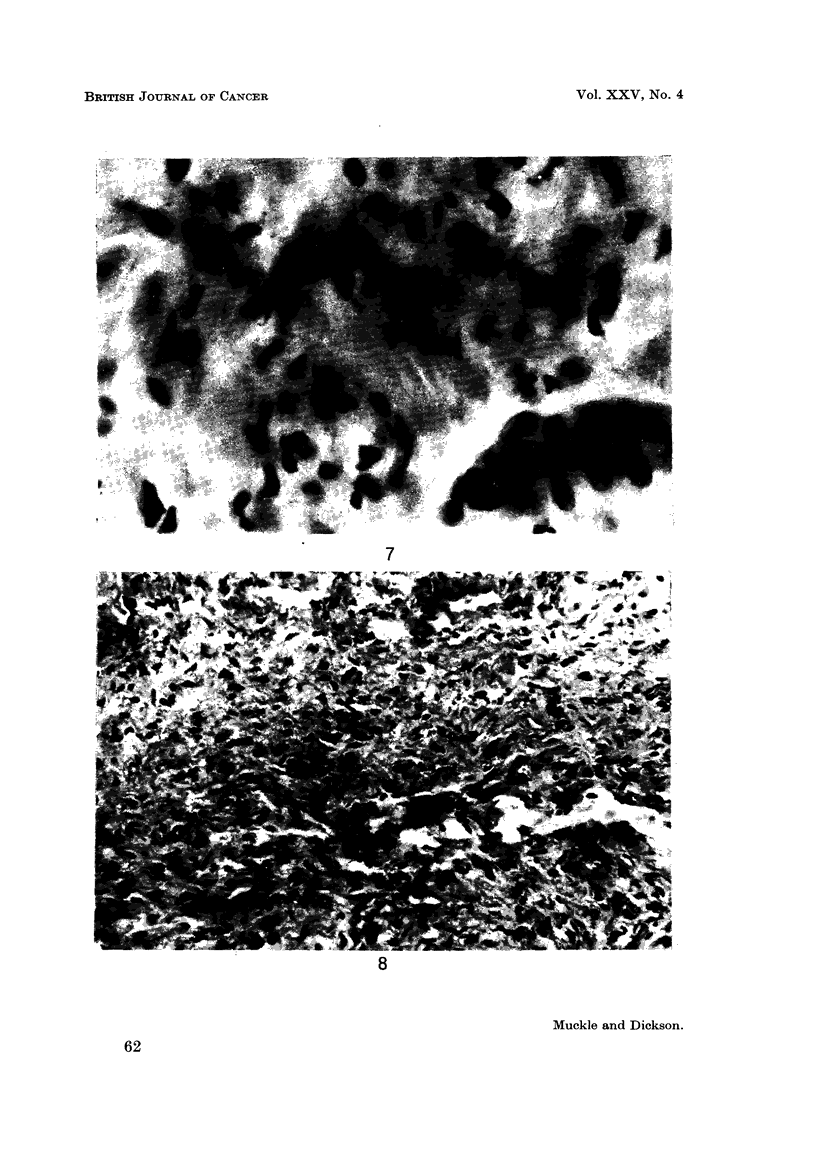

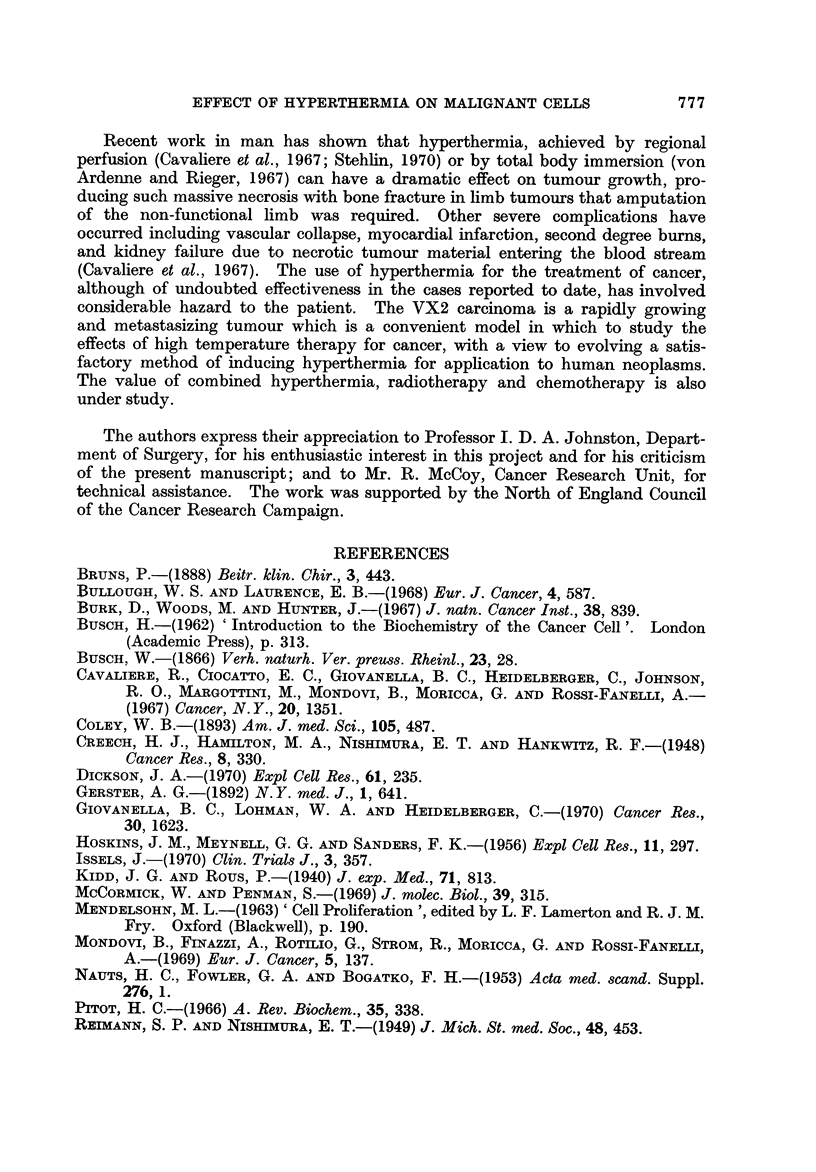

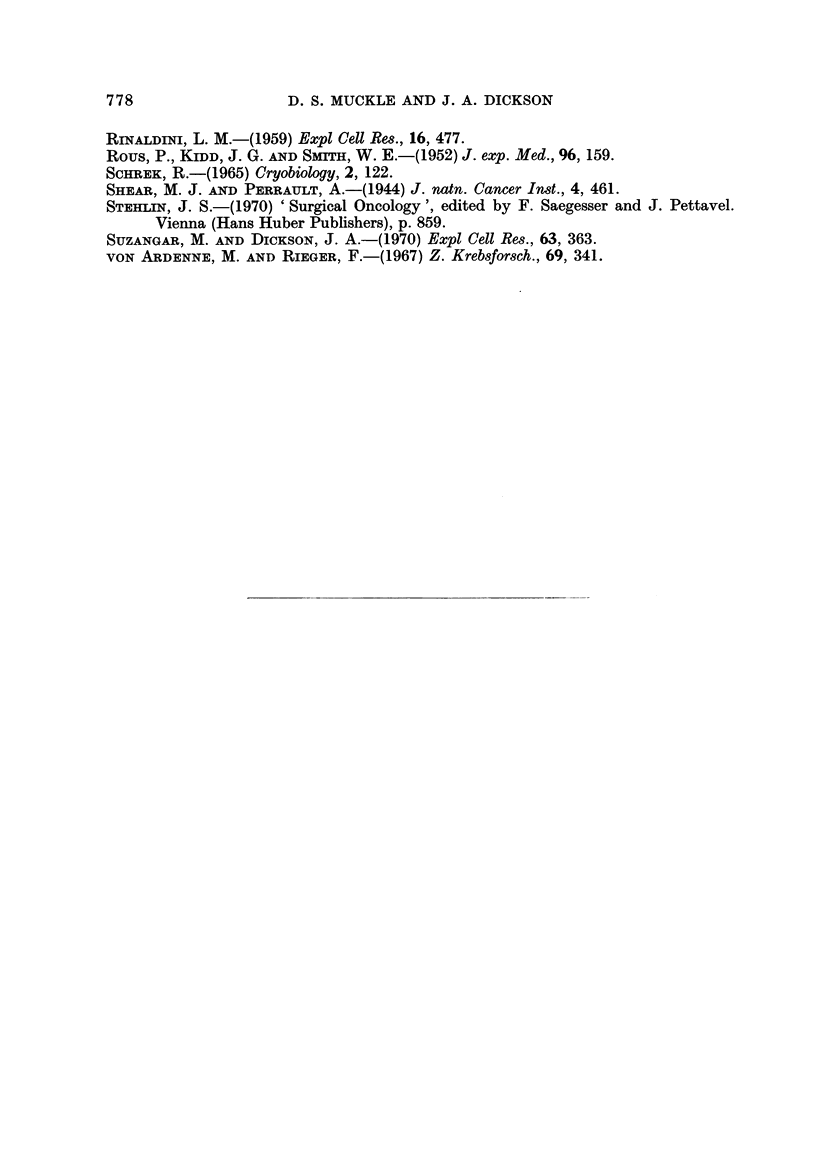


## References

[OCR_00496] Burk D., Woods M., Hunter J. (1967). On the significance of glucolysis for cancer growth, with special reference to Morris rat hepatomas.. J Natl Cancer Inst.

[OCR_00502] Cavaliere R., Ciocatto E. C., Giovanella B. C., Heidelberger C., Johnson R. O., Margottini M., Mondovi B., Moricca G., Rossi-Fanelli A. (1967). Selective heat sensitivity of cancer cells. Biochemical and clinical studies.. Cancer.

[OCR_00513] Dickson J. A. (1970). The uptake of non-metabolizable amino acids as an index of cell viability in vitro.. Exp Cell Res.

[OCR_00518] Giovanella B. C., Lohman W. A., Heidelberger C. (1970). Effects of elevated temperatures and drugs on the viability of L1210 leukemia cells.. Cancer Res.

[OCR_00521] HOSKINS J. M., MEYNELL G. G., SANDERS F. K. (1956). A comparison of methods for estimating the viable count of a suspension of tumour cells.. Exp Cell Res.

[OCR_00525] McCormick W., Penman S. (1969). Regulation of protein synthesis in HeLa cells: translation at elevated temperatures.. J Mol Biol.

[OCR_00531] Mondovì B., Finazzi Agrò A., Rotilio G., Strom R., Moricca G., Rossi Fanelli A. (1969). The biochemical mechanism of selective heat sensitivity of cancer cells. II. Studies on nucleic acids and protein synthesis.. Eur J Cancer.

[OCR_00547] ROUS P., KIDD J. G., SMITH W. E. (1952). Experiments on the cause of the rabbit carcinomas derived from virus-induced papillomas. II. Loss by the Vx2 carcinoma of the power to immunize hosts against the papilloma virus.. J Exp Med.

[OCR_00548] Schrek R. (1965). In vitro methods for measuring viability and vitality of lymphocytes exposed to 45 degree, 47 degree, and 50 degree C.. Cryobiology.

